# Supplementary material for: Light induced non-volatile switching of superconductivity in single layer FeSe on SrTiO3 substrate
Source: Nat Commun. 2019 Jan 8;10:85. doi: 10.1038/s41467-018-08024-w (PMC6325130; doi:10.1038/s41467-018-08024-w)
Supplement: Supplementary file 1 — Supplementary Information [file 41467_2018_8024_MOESM1_ESM.pdf]

SUPPLEMENTARY INFORMATION

Light induced nonvolatile switching of superconductivity in single layer FeSe on $SrTiO_3$ substrate

Yang et al.

Supplementary Figures

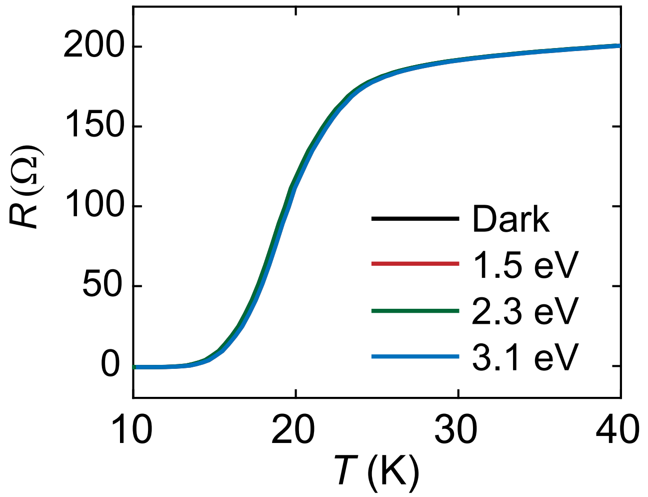

Supplementary Figure 1 Effects of near infrared and visible lights on the superconducting transition. *Temperature dependent resistance of 1 uc FeSe on $SrTiO_3$ (capped by 10 uc FeTe) measured in dark and under three different visible light illuminations with intensities around several mW cm^{-2}.*

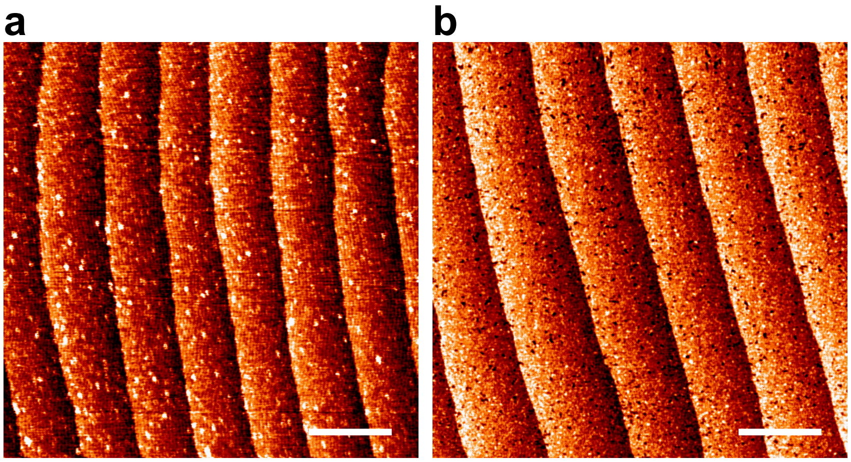

Supplementary Figure 2 STM images of $SrTiO_3$ surface and unannealed monolayer FeSe film *(a) STM image of TiO_2-terminated insulating $SrTiO_3$ (001) substrate surface prior to film growth (V_b=1.9V, I_t=200pA). (b) STM image of 1 ML FeSe/$SrTiO_3$ (001) taken before post annealing (V_b=2.2V, I_t=200pA). The post annealing process removed excessive Se from the FeSe monolayer and also produced small pits in the film as be seen in (b). Scale bars indicate 200 nm.*

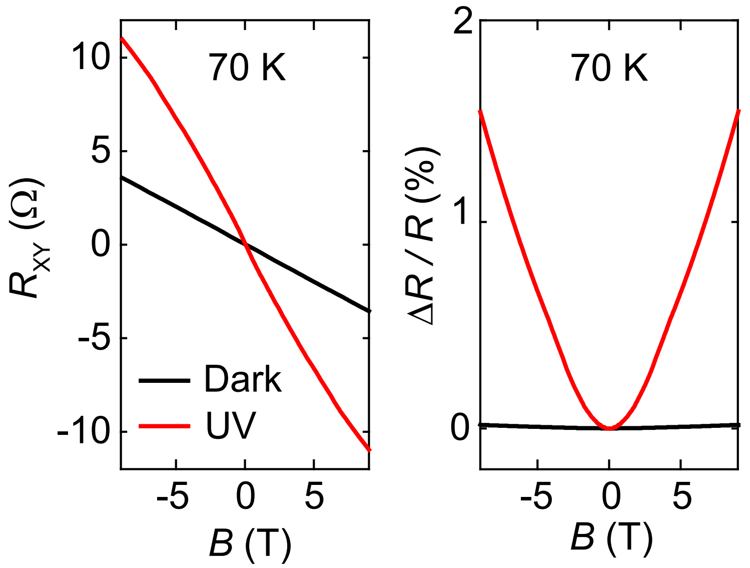

Supplementary Figure 3 UV induced magnetotransport feature change at 70 K

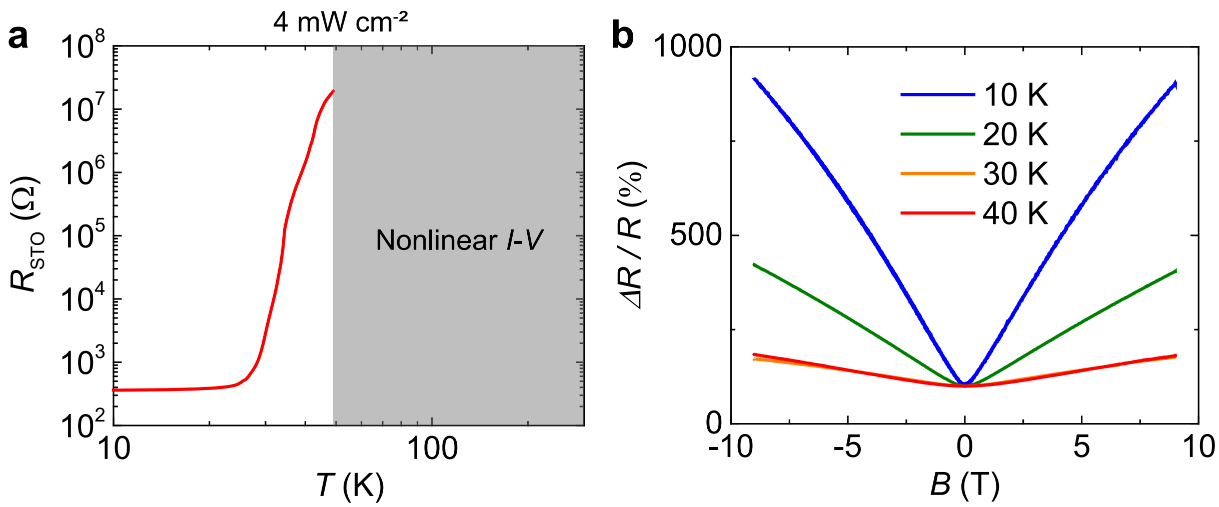

Supplementary Figure 4 UV photoconductance in bare $SrTiO_3$ substrate *(a) Temperature dependent resistance under 4 mW cm^{-2} 3.5 eV UV exposure measured in a $SrTiO_3$ substrate with (001) TiO_2 termination treated by the same method as used in FeSe film growth. Strong Ohmic conductance was observed below 50 K. Without UV light, the sample has a large resistance beyond measurement limit. (b) Magnetoresistance under the same UV exposure measured at low temperatures.*

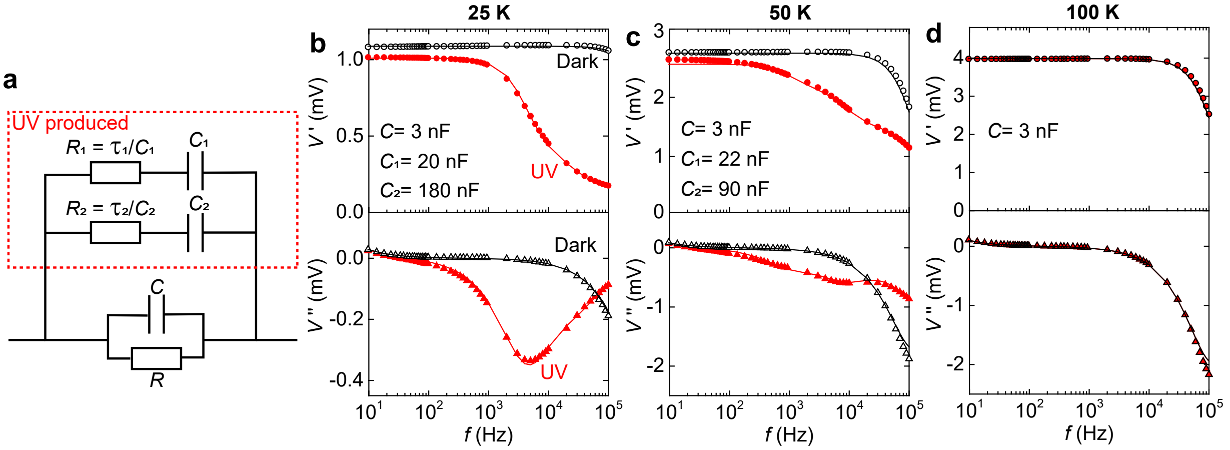

Supplementary Figure 5 UV induced photocapacitance in FeSe/SrTiO$_3$ heterostructure *(a) Circuit model used in the analysis of the impedance spectroscopy data as shown in Figure 4c. Under UV exposure, parallel photocapacitance can be generated from effects such as bulk dielectric properties change, local polarizations, and space charge formations (b-d) Frequency dependences of the in-phase (top) and out-of-phase (bottom) voltage responses measured at 25 K, 50 K, and 100 K. Black circles/ triangles are data measured in dark, and red circles/triangles are data acquired under UV exposure. Solid lines are fitting according to the circuit model as illustrated in (a). During the measurements, a 20 μA AC current was sourced through the sample. At 25 K, although the superconducting transition and reduced resistance in FeSe significantly shunted the parallel impedance signal, a large photocapacitance can still be clearly observed.*

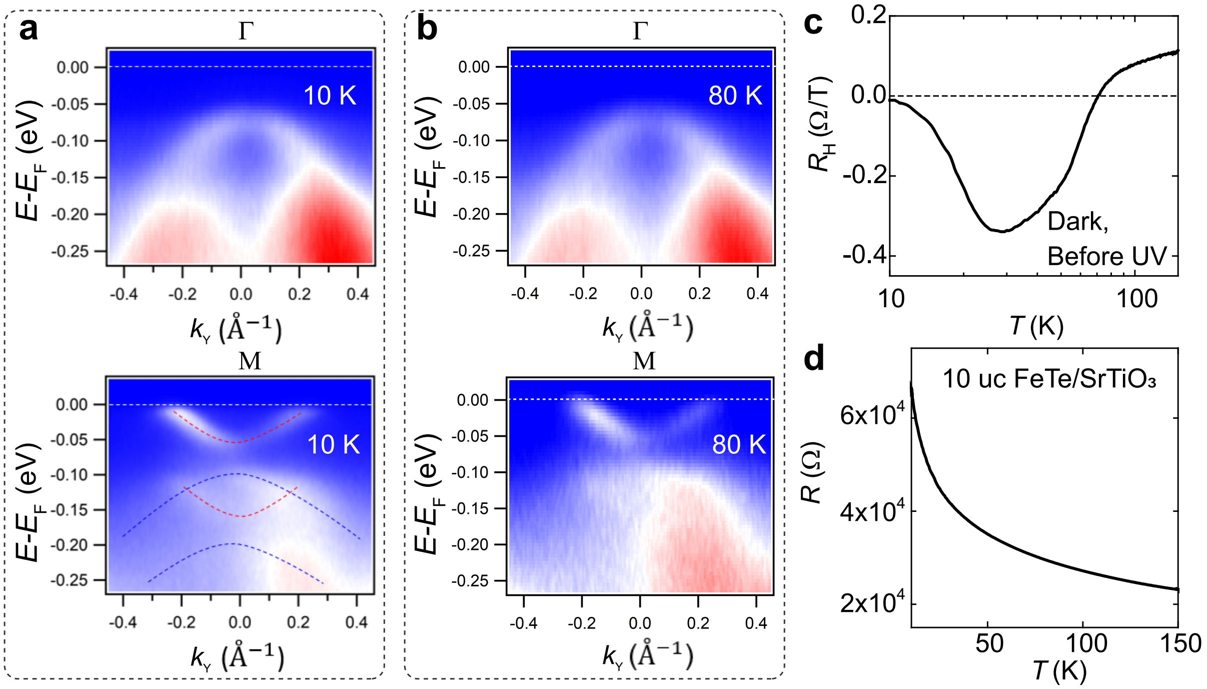

Supplementary Figure 6 Doping level comparison between uncapped and capped sample *(a) In-situ ARPES spectra measured at 10 K on sample S3 (as labeled in Figure 5) without the FeTe capping layer (b) sample measurement obtained at 80 K. The n-type doping in uncapped FeSe was little temperature dependent. (c) Hall resistance measured ex-situ after capping, showing a co-existence of electrons and holes and strong temperature dependence. (d) Resistance of 10 uc FeTe grown directly on* $SrTiO_3$ *measured in identical ways as capped FeSe samples. Resistance of FeTe layer is two orders of magnitude larger than the FeSe samples.*

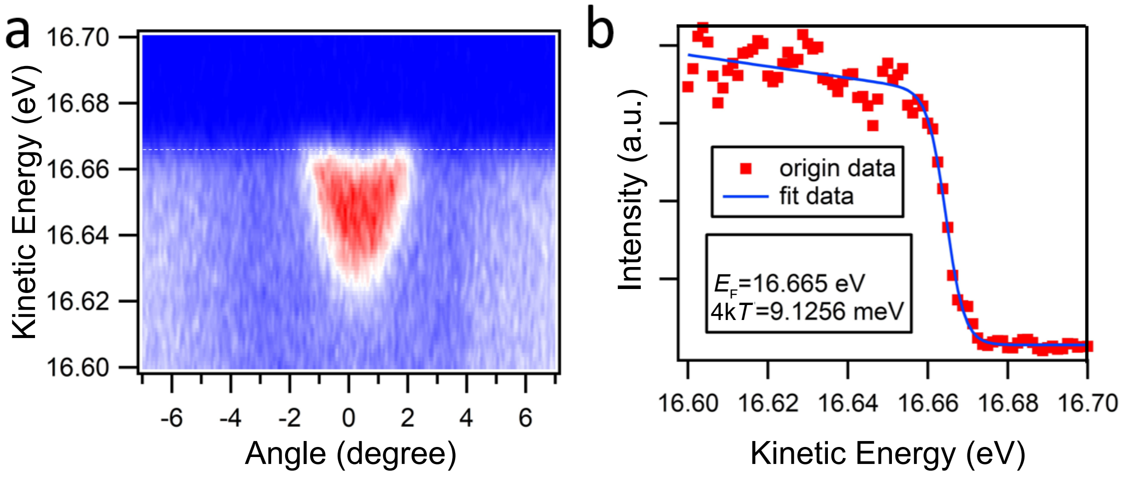

Supplementary Figure 7 ARPES Fermi level calibration by measuring a clean Ag/Si (111) at 10.5 K *(a) Photoemission intensity of Ag/Si (111) by using the angular mode of 14° and pass energy of 10 eV. (b) Plot of energy distribution curve (EDC) of Ag/Si (111). The measured data (red dots) were fitted by Fermi-Dirac function (blue solid line) to determine the Fermi level at 16.665 eV.*

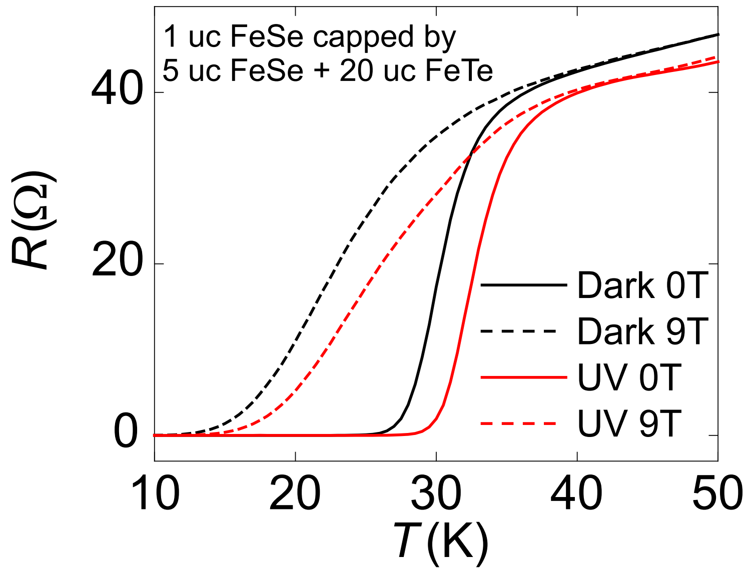

Supplementary Figure 8 Higher superconducting T_C observed in 1 uc FeSe/SrTiO$_3$ with a thicker capping layer. *In this sample, 1 uc FeSe was grown on SrTiO$_3$ using the identical recipe as described in the method section. Instead of directly capping with 10 uc FeTe, another 5 uc FeSe was first deposited layer by layer following the sequence of growing 1 uc FeSe and then annealing it at the growth temperature for 1 hour. Next, a 20 uc FeTe capping layer was deposited before*

the sample was removed from the UHV chamber for subsequent transport measurements. The clearly enhanced T_C *likely resulted from the better shielding of the* $FeSe/SrTiO_3$ *interface from ambient chemical processes and the possible anion intermixing that can occur at the FeSe/FeTe interface.*
